# Preparation of Nanofibrous Structure of Mesoporous Bioactive Glass Microbeads for Biomedical Applications

**DOI:** 10.3390/ma9060487

**Published:** 2016-06-17

**Authors:** Shiao-Wen Tsai, Yu-Han Chang, Jing-Lun Yu, Hsien-Wen Hsu, Lih-Rou Rau, Fu-Yin Hsu

**Affiliations:** 1 Graduate Institute of Biochemical and Biomedical Engineering, Chang-Gung University, Taoyuan 33302, Taiwan; swtsai@mail.cgu.edu.tw (S.-W.T.); elaine_rau12@hotmail.com (L.-R.R.); 2 Department of Orthopaedic Surgery, Chang Gung Memorial Hospital, Linko 33305, Taiwan; yhchang@adm.cgmh.org.tw; 3 Department of Life Sciences and Biotechnology, National Taiwan Ocean University, Keelung 20224, Taiwan; tom081211@yahoo.com.tw (J.-L.Y); s0958292415@gmail.com (H.-W.H.)

**Keywords:** mesoporous bioactive glasses, nanofiber, microbead

## Abstract

A highly ordered, mesoporous (pore size 2~50 nm) bioactive glass (MBG) structure has a greater surface area and pore volume and excellent bone-forming bioactivity compared with traditional bioactive glasses (BGs). Hence, MBGs have been used in drug delivery and bone tissue engineering. MBGs can be developed as either a dense or porous block. Compared with a block, microbeads provide greater flexibility for filling different-shaped cavities and are suitable for culturing cells *in vitro*. In contrast, the fibrous structure of a scaffold has been shown to increase cell attachment and differentiation due to its ability to mimic the three-dimensional structure of natural extracellular matrices. Hence, the aim of this study is to fabricate MBG microbeads with a fibrous structure. First, a sol-gel/electrospinning technique was utilized to fabricate the MBG nanofiber (MBGNF) structure. Subsequently, the MBGNF microbeads (MFBs) were produced by an electrospraying technology. The results show that the diameter of the MFBs decreases when the applied voltage increases. The drug loading and release profiles and mechanisms of the MFBs were also evaluated. MFBs had a better drug entrapment efficiency, could reduce the burst release of tetracycline, and sustain the release over 10 days. Hence, the MFBs may be suitable drug carriers. In addition, the cellular attachment of MG63 osteoblast-like cells is significantly higher for MFBs than for glass microbeads after culturing for 4 h. The nanofibrous structure of MFBs could provide an appropriate environment for cellular spreading. Therefore, MFBs have great potential for use as a bone graft material in bone tissue engineering applications.

## 1. Introduction

Bioactive glasses (BGs), a synthetic silica-based bioactive material, has been widely used in clinical applications since it was first discovered by Larry Hench [1]. Compared to other bioactive ceramics, such as hydroxyapatite and β-tricalcium phosphate, BGs possesses excellent bioactivity and degradation properties [2]. Several studies have found that the release of Ca, P, and Si ions from BGs could stimulate the proliferation and differentiation of osteoblasts and promote bone regeneration.

BGs have been commonly used in either bulk or powder form. Several researchers have demonstrated that cells interact more strongly with nanofibers [3,4,5]. Hence, many studies have focused on the preparation of fibrous BG [6,7,8]. Orefice *et al.* [9] utilized a sol-gel method to prepare bioactive glass fibers. Clupper *et al.* proved that the fiber structure of BG could enhance cellular attachment and spreading [10]. Moreover, Kim found that BG nanofibers could accelerate apatite formation on the surface of the nanofibers in a simulated body fluid (SBF) environment [11].

Highly ordered, mesoporous bioactive glass (MBG) structures (pore size 2~50 nm) have been widely used in bone tissue engineering and drug delivery. Compared with traditional BGs, the large surface area and pore volume of MBGs could greatly enhance bone-forming bioactivity [12,13]. However, most relevant studies are focused on MBGs in powder form, which greatly limits practical applications. Yi *et al.* combined a sol-gel process and a high velocity spray technology to synthesize the microfibers of mesoporous bioactive glasses [14]. Hong *et al.* fabricated the ultrathin mesoporous bioactive glass hollow fibers by using electrospinning combined with a phase-separation-induced agent [15]. Xu *et al.* found that cells had stronger interactions with the nanofibers than microfibers [16]. In a past study, we modified Hong’s method to fabricate mesoporous bioactive glass nanofibers (MBGNFs) and demonstrated the apatite forming ability of MBGNFs in SBF [17]. We found that MBGNFs possess a better drug-loading efficiency and can reduce the burst release of an antibiotic. Additionally, the MBGNFs possessed excellent bioactive properties both *in vitro* and *in vivo* and could be completely adsorbed *in vivo*. Hence, MBGNFs exhibit greater potential as drug carriers and bone substitutes than more traditional materials.

Microbeads can provide a suitable three-dimensional environment for cell growth and differentiation. The more closely packed spherical microbeads also offer greater flexibility for filling different-shaped cavities than nonspherical shapes [18]. Moreover, the empty space between microbeads allows for both new bone and vascular ingrowths. Consequently, microbeads are used as bone filling materials for bone defects with irregular shapes and sizes. Wu [19] fabricated MBG microspheres using a method that combines alginate cross-linking CaCl_2_ with heat treatment, and also proved that MBG microspheres could support cell adhesion and stimulate cell proliferation.

The objective of this study was to fabricate and characterize the nanofibrous structure of mesoporous bioactive glass microbeads (MFBs) by electrospinning and electrospraying methods and to evaluate the release profiles of tetracycline from MFBs along with the cellular behavior on MFBs.

## 2. Materials and Methods

### 2.1. Reagents

Pluronic P123 (MW = 5800), tetraethyl orthosilicate (TEOS), triethyl phosphate (TEP), calcium nitrate tetrahydrate, poly(vinyl pyrrolidone) (PVP), and sodium alginate were purchased from Sigma-Aldrich Chemical Company (St. Louis, MO, USA). All other chemicals used were reagent grade, unless otherwise stated.

### 2.2. Synthesis of MBGNFs

The method for fabricating the MBGNFs has been described previously [17]. Briefly, the MBG precursor solution (molar ratio of Si/Ca/P = 78.8:14.4:6.8) was prepared by mixing Pluronic P123 (1.0 g), TEOS (1.59 mL), calcium nitrate tetrahydrate (0.315 g), TEP (99.8%, 0.114 mL), hydrochloric acid (1 M, 0.804 mL), and ethanol (10 mL). Subsequently, Pluronic P123 (0.2 g), PVP (0.5 g) and ethanol (2.5 mL) were prepared and added into the MBG precursor solution (5 mL) to obtain a MBGNF precursor solution. The MBGNF precursor solution was then drawn into a plastic syringe with an 18G needle and placed into a syringe pump (RAZEL, Model R99-E), which supplied a steady flow rate at 763 µL/h. A high electric field (1.0 kV/cm) was applied between the needle tip and grounded collector. The non-woven nanofibers on the grounded collector were collected and calcined at 600 °C for 5 h to eliminate the organic components and produce the MBGNF nonwoven structures The diameters of the fibers were measured by randomly choosing 100 fibers from SEM images. ImageJ (ImageJ software 1.42, National Institutes of Health, Stapleton, NY, USA) was used to analyze the samples. Data are expressed as mean ± standard deviations.

### 2.3. Preparation and Characterization of the MBGNF Microbeads

The microbeads were prepared using a method of alginate crosslinking with a calcium chloride solution and an electrostatic droplet generator [20]. The calcined MBGNF nonwoven structure was reduced to fragments by a sonicator. The MBGNF fragments (40 mg) were added into a 1-mL water solution and stirred for 30 min to form a slurry. Sodium alginate powder was dissolved in water to form the alginate solution with a concentration of 4% (*w*/*v*). The 1-mL alginate solution was added to 1 mL of the MBGNF slurry and ultrasonicated to form the mixture solution. The mixture solution was dropped into a 1.5% CaCl_2_ (pH 7.2) solution through a syringe equipped with a needle-cannula (21G), and the high-voltage electrostatic system was then employed to form the MBGNF/alginate gel beads. Subsequently, the MBGNF/alginate gel beads were calcined at 600 °C for 6 h to obtain the MBGNF microbeads (MFBs).

The diameters of the MFBs were analyzed by an image analysis program (ImageJ software 1.42, National Institutes of Health, Stapleton, NY, USA). The surface morphology of the MFBs was examined by scanning electron microscopy (SEM). The MFBs were embedded in resin and ultramicrotomed, and the microstructure of the MFBs was then analyzed using transmission electron microscopy (TEM). The Brunauer–Emmett–Teller (BET) specific surface area and pore size were obtained from nitrogen adsorption isotherms measured at −196 °C using an ASAP 2020 instrument (Micromeritics, Norcross, GA, USA). The average pore size was calculated using the Barrett–Joyner–Halenda (BJH) method.

### 2.4. In Vitro Study of Drug Loading and Release

Tetracycline (TC) was selected as the model drug for drug loading and release evaluation. Ten milligrams of MFBs was added into 1 mL of a TC solution (5 mg/mL) and stirred for 24 h at 37 °C. After the loading procedure, the amount of TC adsorbed on the MFBs was calculated by determining the difference in the initial and final concentration of TC in the supernatant. The concentration of TC was measured using an UV-Vis spectrophotometer at a wavelength of 360 nm. The drug contents and loading efficiency were calculated as follows:

Drug contents (*w*/*w*) = weight of drug in MFB/weight of MFB (1)

Entrapment efficiency (%) = (weight of drug in MFB/weight of drug fed initially) × 100%
(2)

After loading, the MFBs were lyophilized. The TC-loaded MFBs were placed into 10 mL of a PBS (pH 7.2) solution and agitated in a horizontally shaking water-bath maintained at 37 °C. The release medium was withdrawn every day and replaced with a fresh medium at each measurement. The concentration of TC in the PBS was analyzed. The rate and mechanism of TC release from the MFBs were analyzed by fitting the release data into various kinetic models. These models were zero-order equation (*Q*_t_ = *k*_0_*t*), first-order equation (ln*Q*_t_ = ln*Q*_0_ − *k*_1_*t*), and Fickian matrix diffusion for spheres (*Q*_t_ = *k*_f_*t*^0.43^), where Q is the cumulative amount of drug released at time *t*, *Q*_0_ is the initial amount of drug in the microbeads. *k*_0_, *k*_1_, and *k*_f_ are the rate constants of zero order, first order, and Fickian equations, respectively [21]. The release data were further analyzed by the Ritger–Peppas equation (*M*_t_/*M*_∞_ = *k*_r_*t*^n^), where *M*_t_ is the amount of drug released at time *t*, and *M*_∞_ is the amount of drug released at time ∞. The parameters *k*_r_ and *n* are the release rate constant and release exponent, respectively.

### 2.5. Cellular Adhesion on MFB

The osteoblast-like cells (MG63, BCRC No. 60279, Bioresource Collection and Research Center, Taipei, Taiwan) were maintained in a modified Eagle’s medium with 10% fetal bovine serum, 50 µg/mL ascorbic acid, 10 mM *ß*-glycerophosphate, 100 U/mL penicillin, and 100 μg/mL streptomycin. The MFBs and glass microbeads (425~600 µm, Sigma-Aldrich, St. Louis, MO, USA, used as a control material) were placed in 3-cm tissue culture dishes, and the MG63 cells (1 × 10^5^ cells/well) were seeded onto the microbeads. The cell-seeded microbeads were harvested after 1 day to evaluate the cellular adhesion.

### 2.6. Cellular Morphology

The cellular morphology was analyzed by SEM. The microbeads were washed with PBS, and then fixed with glutaraldehyde solution (2.5%) for 1 h. After washing in PBS, the microbeads were post-fixed in osmium tetroxide solution (1%) for an additional hour. The fixed microbeads were dehydrated in a series of gradient ethanol solutions. The dehydrated microbeads were critical-point dried, sputter coated with gold, and then observed using SEM.

### 2.7. Statistical Analyses

Results are expressed as mean ± standard deviation. For each condition, at least 50 microbeads were chosen randomly to measure diameter of microbeads. The microbeads diameter was analyzed using a one-way ANOVA with Tukey’s *post hoc* test. Differences at *p* < 0.05 were considered statistically significant.

## 3. Results and Discussion

**Figure 1:**
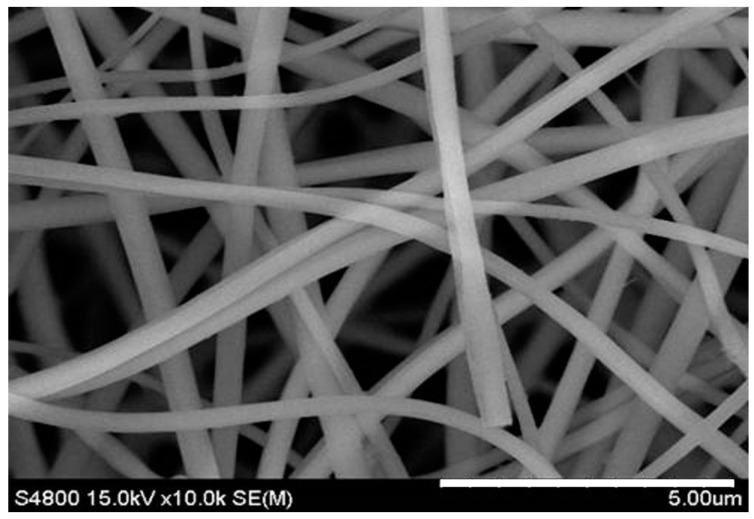
Scanning electron microscopy (SEM) images of mesoporous bioactive glass nanofibers (MBGNFs).

The surface morphology of the MBGNF was examined with SEM (Figure 1). The MBGNF fiber diameter was 276 ± 58 nm. This dimension is similar to that of the native fibrous protein in an extracellular matrix.

**Figure 2:**
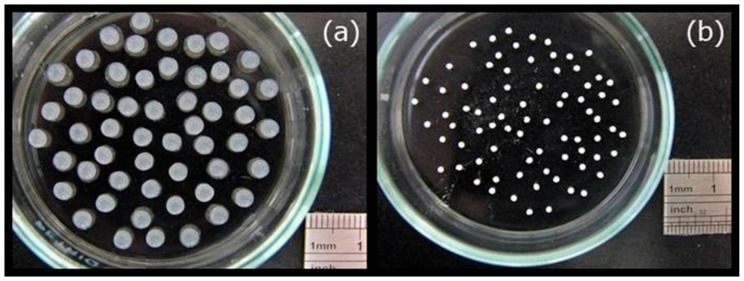
Optical images of (**a**) MBGNF/alginate gel beads and (**b**) MFBs.

**Figure 3:**
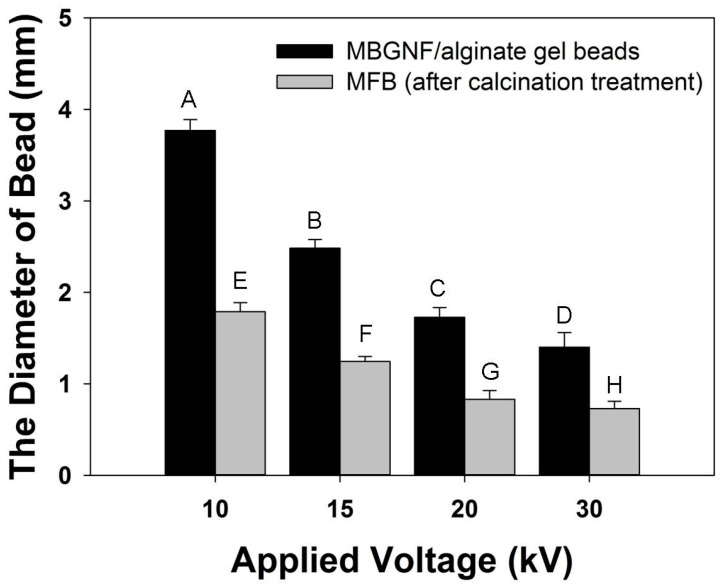
The diameter of MBGNF/alginate gel beads and MFBs as a function of the voltage magnitude. The data are presented as mean ± standard deviation, *n* = 50. The different letters represent significance at *p* < 0.05, as determined by the one-way ANOVA with Tukey’s *post hoc* test.

**Figure 4:**
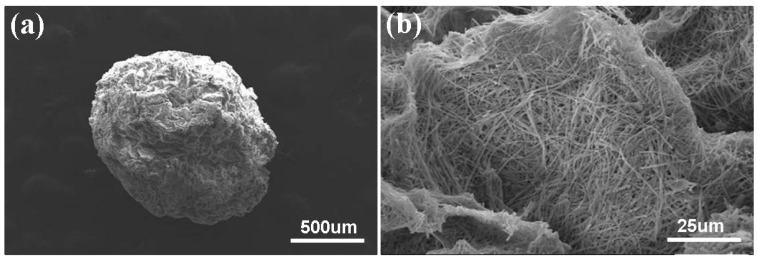
SEM images of a MFB surface in different magnifications (**a**) ×50, scale bar = 500 μm and (**b**) ×1000, scale bar = 25 μm.

**Figure 5:**
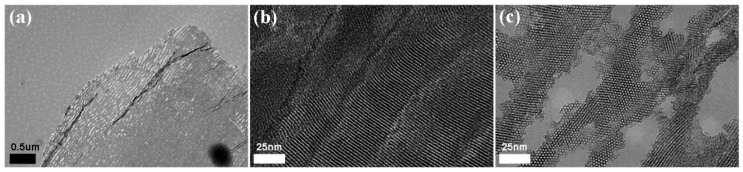
TEM micrograph of the ultramicrotomed MFB embedded in resin. The microstructure of the MFB retained (**a**) a nanofibrous structure; (**b**) long one-dimensional channels; and (**c**) a highly ordered 2D hexagonal structure. The magnification of (**a**) ×10 k, scale bar = 0.5 μm; (**b**,**c**) ×250 k, scale bar = 25 nm.

A digital camera was used to obtain photographs (Figure 2) of the morphology of the MBGNF/alginate gel beads and the MFB. The magnitude of the voltage generated between the needle and the gelling bath are important variables in determining droplet size. The applied potential and gel bead diameter are inversely related (Figure 3). When the applied potential was increased from 10 to 30 kV, the diameter of the MBGNF/alginate gel beads decreased from 3.8 to 1.4 mm. The diameter of the MFBs varied from 1.8 to 0.7 mm after the calcined treatment. The topography of MFBs was examined with SEM and TEM. The SEM images showed that the nanofibers in the MFB microstructure (as shown in Figure 4a,b) remained intact, as did the ordered one-dimensional channels of the hexagonally packed mesostructure of a resin embedded, ultramicrotomed MFB (shown in the TEM micrograph, Figure 5a,b).

**Figure 6:**
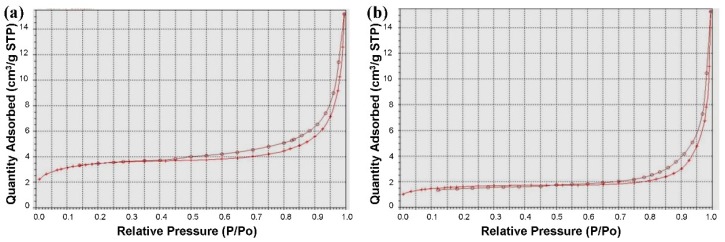
N_2_ adsorption–desorption isotherm of (**a**) MBGNFs and (**b**) MFBs.

The nitrogen sorption isotherms of the MBGNFs and MFBs are type IV hysteresis loops, which are typical for mesoporous materials with one-dimensional cylindrical channels (Figure 6). The pore sizes of the MBGNFs and the MFBs are 3.9 nm and 3.7 nm, respectively. The BET surface areas of the MBGNFs and MFBs are 29.71 and 11.20 m^2^/g, respectively. After calcined treatment, both the pore size and surface area of the MFBs are smaller than the MBGNFs.

**Figure 7:**
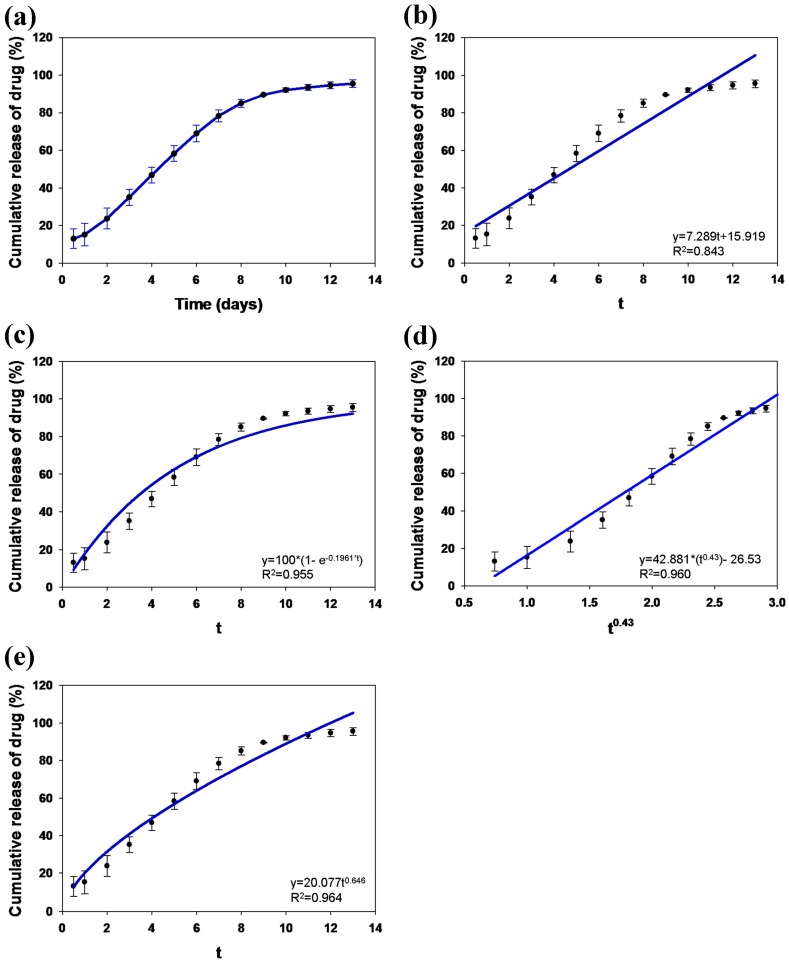
(**a**) *In vitro* cumulative tetracycline (TC) release from TC-loaded MFB. Curve was fitted with an equation of (**b**) zero-order; (**c**) first-order; (**d**) Fickian matrix diffusion; (**e**) Ritger-Peppas models.

The amount of loaded TC within the MFBs was 4.72 ± 0.07 mg/10 mg (TC/MFB). The entrapment efficiency of TC was 94.36% ± 1.50% (*w*/*w*). The cumulative drug release profile for TC release from the TC-loaded MFB as a function of time was measured in triplicate (Figure 7a). The drug release data indicates that the MFB could reduce the burst release over the first 24 h compared with other forms of MBG [22]. Moreover, the MFB exhibited a prolonged release of TC over 10 days. The drug release data were fitted to equations of zero-order (Figure 7b), first-order (Figure 7c), and Fickian matrix diffusion (Figure 7d) for spheres, to evaluate the drug release mechanism and kinetics. The best fit with the highest correlation coefficient (*r*^2^) was obtained using the Fickian matrix diffusion for spheres (*r*^2^ = 0.960), followed by the first-order equation (*r*^2^ = 0.955) and then the zero-order equation (*r*^2^ = 0.843). The release data were also analyzed using the Ritger–Peppas model (Figure 7e) [23]. According to this model, the release exponent *n*-value was 0.646, suggesting that the drug release followed both diffusion and erosion-controlled mechanisms [24].

**Figure 8:**
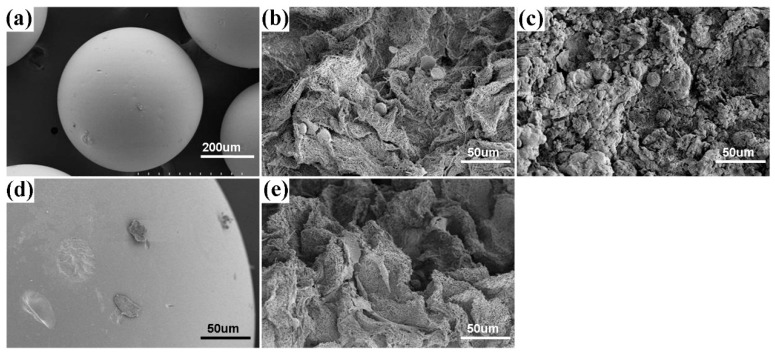
Representative images of the MG63 osteoblast-like cells cultured on microbeads for 1 and 3 d. (**a**,**d**) GBs; (**b**,**e**) MFBs; (**c**) MBs; (**a**–**c**) 1 day; (**d**,**e**) 3 days.

Cell attachment and spreading are also known to be critically sensitive to the surface topography and the molecular composition of the matrix. To evaluate the effect of surface topography of the mesoporous bioactive glass on cell behavior, we incubated MG63 osteoblast-like cells on the glass beads (GBs), the mesoporous bioactive glass beads (MBs) and the MFBs. The results, shown in Figure 8a,b, indicate that the attachment of the MG63 osteoblast-like cells is significantly higher on the MFBs and MBs than on the GBs after 1 day of culture. The SEM data indicate that the cell shape remained circular on the MBs (Figure 8c), while cell spreading had occurred on the MFBs (Figure 8b). The morphology of the MG63 osteoblast-like cells cultured on the MFBs exhibited a polygonal shape with lamellipodia (Figure 8d) and a round shape on the GBs after 3 day of culture (Figure 8e), whereas the MBs gradually collapsed into small pieces during the cell incubation process.

## 4. Conclusions

In this work, we have successfully fabricated the nanofibrous structure of mesoporous bioactive glass microbeads using electrospinning and electrospraying technology. The nanofibrous structure of the microbeads provided an excellent environment for MG63 osteoblast-like cell attachment and spreading. Moreover, the MBGNF microbeads possessed excellent drug loading ability and exhibited a prolonged release of tetracycline over 10 days. Hence, the MBGNF microbeads have the potential to be a novel bone graft substitute for bone regeneration.
